# Assembly and comparative genome analysis of four mitochondrial genomes from *Saccharum* complex species

**DOI:** 10.3389/fpls.2024.1421170

**Published:** 2024-07-19

**Authors:** Sicheng Li, Cuifang Yang, Zhen Wang, Chaohua Xu, Gemin Zhang, Yuxin Huang, Baoqing Zhang, Shan Zhou, Yijing Gao, Wenyi Zong, Weixing Duan, Xiping Yang

**Affiliations:** ^1^ Sugarcane Research Institute, Guangxi Academy of Agricultural Sciences/Guangxi Key Laboratory of Sugarcane Genetic Improvement/Key Laboratory of Sugarcane Biotechnology and Genetic Improvement (Guangxi), Ministry of Agriculture & Rural Affairs, Nanning, China; ^2^ State Key Laboratory of Conservation and Utilization of Subtropical Agro-Bioresources, Guangxi University, Nanning, China; ^3^ Guangxi Key Laboratory of Sugarcane Biology, Guangxi University, Nanning, China; ^4^ National Key Laboratory for Biological Breeding of Tropical Crops, Sugarcane Research Institute, Yunnan Academy of Agricultural Sciences, Kunming, China

**Keywords:** mitochondrial genome, phylogenetic, Saccharinae, *Tripidium arundinaceum*, *Erianthus rockii*, *Miscanthus sinensis*, *Narenga porphyrocoma*

## Abstract

*Saccharum* complex includes genera *Saccharum*, *Miscanthus*, *Erianthus*, *Narenga*, and *Tripidium*. Since the *Saccharum* complex/Saccharinae constitutes the gene pool used by sugarcane breeders to introduce useful traits into sugarcane, studying the genomic characterization of the Saccharum complex has become particularly important. Here, we assembled graph-based mitochondrial genomes (mitogenomes) of four Saccharinae species (*T. arundinaceum*, E. rockii, *M. sinensis*, and *N. porphyrocoma*) using Illumina and PacBio sequencing data. The total lengths of the mitogenomes of *T. arundinaceum*, *M. sinensis*, *E. rockii* and *N. porphyrocoma* were 549,593 bp, 514,248 bp, 481,576 bp and 513,095 bp, respectively. Then, we performed a comparative mitogenomes analysis of Saccharinae species, including characterization, organelles transfer sequence, collinear sequence, phylogenetics analysis, and gene duplicated/loss. Our results provided the mitogenomes of four species closely related to sugarcane breeding, enriching the mitochondrial genomic resources of the Saccharinae. Additionally, our study offered new insights into the evolution of mitogenomes at the family and genus levels and enhanced our understanding of organelle evolution in the highly polyploid *Saccharum* genus.

## 1 Introduction

In the Andropogoneae tribe, there are seed-based commercial crops such as maize, sorghum, and *Coix lacryma-jobi* (Wall and Ross, 1970; Ranum et al., 2014; Xi et al., 2016) and leaf-based commercial crops like *Chrysopogon zizanioides* (Chahal et al., 2015). Additionally, crops like sugarcane, which primarily yield from their stalks, are of significant commercial importance (Bell and Garside, 2005). Sugarcane belongs to the genus *Saccharum* and is the principal constituent of the *Saccharum* complex, which also includes other genera such as *Miscanthus*, *Erianthus*, *Narenga*, *Sclerostachya*, and *Tripidium* (Vasquez et al., 2022). Some of these genera are clearly not closely related to *Saccharum* (Li et al., 2022). Indeed, recent phylogenetic studies indicate that *Tripidium* is over 11 million years divergent from *Saccharum*, and *Eriochrysis* is even more divergent (Evans and Joshi, 2020). *Miscanthus* species are traditionally believed to possess a basic chromosome number of n = x = 19, and genome sequencing studies have shown that *Miscanthus sinensis* is a paleotetraploid comprising the A and B subgenomes (Mitros et al., 2020). Initially considered the closest diploid relative of sugarcane, *Narenga porphyrocoma* (2n = 30) has been estimated to have diverged from sugarcane approximately 2.5 million years ago (Evans and Joshi, 2020). *Erianthus rockii* (2n = 4x = 30) is a drought- and cold-tolerant wild relative of sugarcane from China (Qi et al., 2022).

To broaden the genetic base of modern sugarcane cultivars, sugarcane breeders aim to enrich the sugarcane gene pool through intergenic crosses with sugarcane relatives, enhancing yield, stress resistance, and disease resistance. Sugarcane breeders have used species from closely related genera to improve sugarcane varieties. *Tripidium*, for example, has demonstrated considerable cold hardiness and biomass yields (Mahadevaiah et al., 2023); *N. porphyrocoma* has excellent characteristics such as high tillering ability, drought tolerance, and mosaic disease resistance (Liu et al., 2018). Wild-type clones of *Tripidium arundinaceum* and *Saccharum spontaneum* show the potential to provide resistance to smut and high biomass, fiber, and bioenergy (Anna Durai and Karuppaiyan, 2023; Meena et al., 2024). Given that the *Saccharum* complex/Saccharinae serves as the gene pool for sugarcane breeders attempting to introgress useful traits into sugarcane, determining the phylogenetic relationships among these genera and species through molecular strategies is of considerable significance and relevance.

Mitochondria are essential for cellular energy production and numerous biological processes, including growth, development, and adaptation to environmental stress, potentially affecting agronomic traits (Mahadevaiah et al., 2023). However, the specific impact may vary depending on plant species and environmental conditions. Plant mitochondria are more complex and have a higher proportion of non-coding regions compared to animal mitochondria (Møller et al., 2021), likely because plants must manage various biotic and abiotic stresses to maintain proper physiological functions. Given their role as the cell’s energy supply system and their predominantly matrilineal inheritance in most plants (Li et al., 2021), mitochondria are an excellent research focus for studying parental relationships in polyploidization and the evolution of the energy supply system (Vallejo-Marín et al., 2016).

Recent studies have identified different conformations of mitochondrial genomes (mitogenomes), including linear structure of *Lactuca sativa* (Guo et al., 2019), branched structure of some cytoplasmic male sterility (CMS) lines in *Zea mays* (Zhang et al., 2020b), and numerous circular arrangements, such as *Silene noctiflora* (Wu et al., 2015) and *Ombrophytum subterraneum* (Roulet et al., 2020). Mitogenomes in the Poaceae family usually consist of individual ring DNA molecules, as in *Oryza minuta* (Asaf et al., 2016), *Sporobolus alterniflorus* (Wang et al., 2021), *Avena longiglumis* (Liu et al., 2023), *Elymus magellanicus* (Chen et al., 2023b), and *Eleusine indica* (Hall et al., 2020). However, there are two distinct circular DNA molecules in the mitogenome of *Saccharum* species (Evans et al., 2019). With advances in sequencing technology and assembly strategies, the master circular model no longer fully characterizes the actual mitochondrial genome.

Currently, only the mitogenomes of *Saccharum* spp. have been published (Li et al., 2024), leaving numerous properties of Saccharinae mitogenomes yet to be discovered. In this research, we *de novo* assembled the complete mitogenome of four Saccharinae species (*T. arundinaceum*, *M. sinensis*, *E. rockii*, and *N. porphyrocoma*) using a combination of second-generation Illumina sequencing and third-generation PacBio sequencing technologies. Then, mitogenome organization, characteristic, phylogenetic relationship, and comparative genome analyses were performed. Our study revealed reticulate mitochondrial conformations featuring multiple junctions. By comparing the organellar genomes of four Saccharinae species, we aimed to identify structural, sequence, and evolutionary differences within Saccharinae that have significant contributions to the cultivar enhancement of *Saccharum* cultivars.

## 2 Materials and methods

### 2.1 Plant materials and genome sequencing

Four accessions from the *Saccharum* complex, including BM87-36 (*T. arundinaceum*), M022 (*M. sinensis*), DZM1 (*E. rockii*), and HBW017 (*N. porphyrocoma*), were used for mitogenome investigation (**Table 1**). Leaf samples of the four accessions were collected (September 1, 2023) in Guangxi Key Laboratory of Sugarcane Genetic Improvement (22°50′N, 108°15′E). The extracted total genomic DNA was used for library construction with 150-bp and 15-kb insert sizes and then sequenced on the Illumina NovoSeq 6000 sequencing platform (Illumina, San Diego, CA, USA) and PacBio Revio platform for short and long reads, respectively. Finally, the Illumina and Nanopore high-quality reads were obtained and processed (**Supplementary Table S1**).

**Table 1:** Sample information of *Tripidium arundinaceum*, *Miscanthus sinensis*, *Erianthus rockii*, and *Narenga porphyrocoma*.

Sample	Species	Region	Chromosome number
BM87-36	*T. arundinaceum*	Guangxi, China	2n = 60
DZM1	*E. rockii*	Guangxi, China	2n = 30
M022	*M. sinensis*	Guangxi, China	2n = 38
HBW017	*N. porphyrocoma*	Guangxi, China	2n = 30

### 2.2 Mitogenome assembly and annotation

A hybrid assembly strategy was used for mitogenome assembly. First, the GetOrganelle (v1.7.6.1) was used to assemble short reads into a corresponding unitig graph with the parameters ‘-R 30 -k 85,105,115,127 -F embplant_mt’, and the contigs that contained the mitochondrial core genes in Andropogoneae were selected. The mitochondrial contigs obtained by GetOrganelle assembly were then used as bait to extract mitochondrial PacBio HiFi reads by Seqkit (v2.2.0); then, they were assembled by flye (2.9.1-b1780) with the parameters ‘–pacbio-hifi –meta -g 500K -t 20’; and the final mitogenome was visualized and adjusted manually by the Bandage (v0.8.1) software (Wick et al., 2015).

The mitogenome of four Saccharinae species was annotated by Geseq (https://chlorobox.mpimp-golm.mpg.de/geseq.html) and IPMGA (http://www.1kmpg.cn/mgavas/) and then manually annotated and corrected based on the reference mitochondrial genome of *Sorghum bicolor* and *Saccharum officinarum* (Khon Kaen 3) [National Center for Biotechnology Information (NCBI) accession numbers: NC008360.1 and NC031164.1]. The mitogenome structure map was drawn using OGDRAW (http://ogdraw.mpimp-golm.mpg.de/cgi-bin/ogdraw.pl).

### 2.3 Validation of linkage junction and PCR amplification

Linkages between graphical contigs were designed within a range of 100–500 bp on either side of each junction site using the Primer 3 program (http://bioinfo.ut.ee/primer3-0.4.0/) (**Supplementary Table S2**). The DNA isolated from young leaf tissue of each species was used to conduct PCR verification. PCR was carried out in a 20-µL reaction mixture containing 10 µL of 10× reaction buffer, 5 pmol of each primer, 1.25 units of Taq DNA polymerase, and 20 ng of DNA template. The PCR was performed in thermocyclers using the following cycling parameters: 94°C (5 min); 30 cycles of 94°C (30 s), 55°C–57°C (30 s); 72°C (30 s), then 72°C (7 min). PCR products were visualized on agarose gels (2.0%–3.0%) containing Safe gel stain.

### 2.4 Organelle DNA sequence transformation

Transfer fragments between mitogenome and chloroplast genome (MTPTs) and between mitogenome itself (MTMTs) were identified by Blastn (2.5.0+) (parameters: e-value 1e^−10^ -outfmt 6). The Circos plot was visualized using the Advanced Circos module in TBtools (Chen et al., 2023a).

### 2.5 Phylogenetic analysis

To better and comprehensively explore the evolutionary relationship of the *Saccharum* complex, the 12 mitogenomes of Poaceae (**Supplementary Table S3**) were downloaded from the NCBI database. A total of 13 shared protein-coding genes (PCGs) among the analyzed species were identified and extracted using PhyloSuite (v1.2.2) (Zhang et al., 2020a). All the PCGs were aligned in batches with MAFFT (v7.313) (Katoh and Standley, 2013) and integrated into PhyloSuite using normal-alignment mode. Maximum likelihood phylogenies were inferred using IQ-TREE under the Edge-unlinked partition model for 50,000 ultrafast bootstraps, and the tree was visualized using iTOL.

### 2.6 Collinear analysis and comparative genome analysis

Six species related and within *Saccharum* complex were selected for analysis, including *S. bicolor* (NC013816.1), *T. arundinaceum* (in this study), *M. sinensis* (in this study), *E. rockii* (in this study), *N. porphyrocoma* (in this study), and *S. spontaneum* (Li et al., 2024), to conduct comparative mitogenome analysis and collinearity analysis. To investigate the similarity of mitogenome sequences within the *Saccharum* complex and closely related species, homologous sequences between the four relatives were detected using Blastn (2.5.0+) (parameters: e-value 1e^−10^). Homologous sequences less than 0.5 kb were not retained. A multiple synteny plot of those four mitogenomes was generated using TBtools.

A dot plot of pairwise comparison on conserved collinear blocks was generated and plotted using MUMmer (Kurtz et al., 2004). Based on sequence similarity, a Multiple Synteny Plot of the five mitogenomes from this and previous studies with closely related species was plotted using MCScanX in TBtools.

## 3 Results

### 3.1 Mitogenome assembly, annotation, and gene features

**Figure 1:**
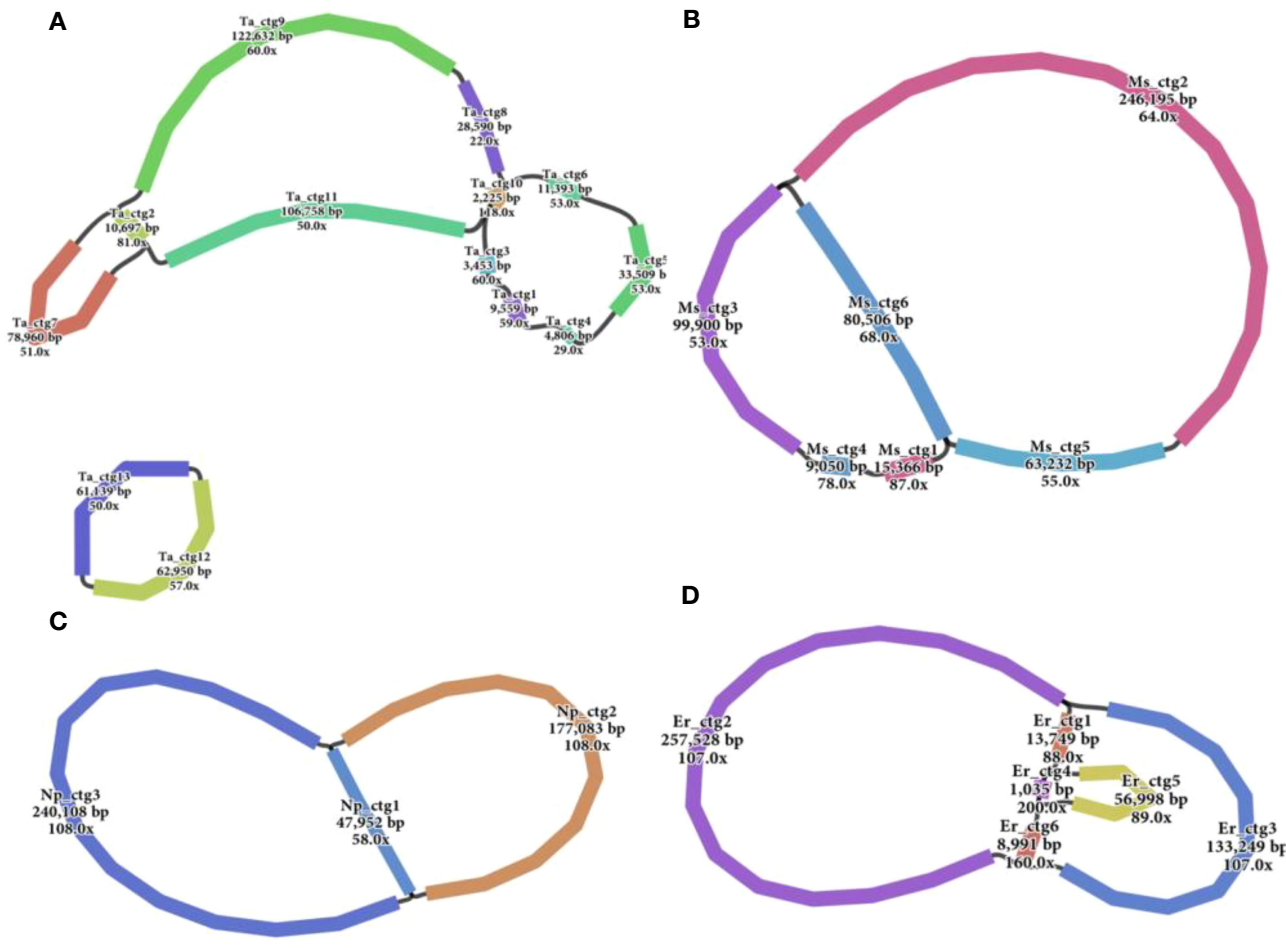
Branched conformation of four Saccharinae species mitogenomes. **(A)** *Tripidium arundinaceum*, **(B)** *Miscanthus sinensis*, **(C)** *Narenga porphyrocoma*, and **(D)** *Erianthus rockii*.

**Figure 2:**
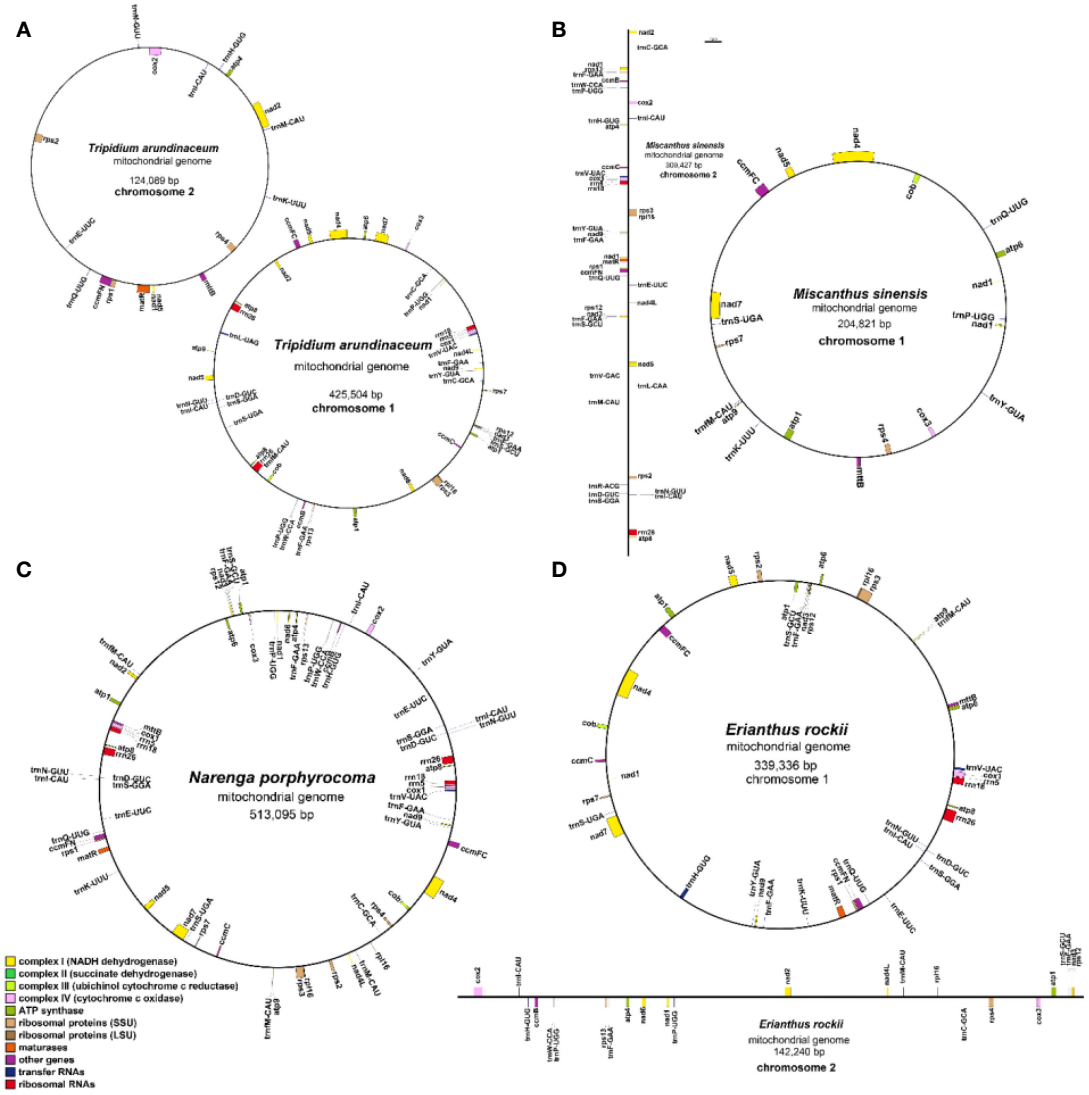
Gene map of the four Saccharinae species mitogenomes. **(A)** *Tripidium arundinaceum*, **(B)** *Miscanthus sinensis*, **(C)** *Narenga porphyrocoma*, and **(D)** *Erianthus rockii*.

Accurate mitogenomes were obtained by combining Illumina and PacBio HiFi reads. Consistent depths of mapping reads revealed the high-quality gap-free assembly (**Supplementary Table S4**). First, the mitogenome of four *Saccharum* complex species was assembled into initial graph-based structures. From PacBio data, 13 contigs of *T. arundinaceum*, six contigs of *M. sinensis*, three contigs of *N. porphyrocoma*, and six contigs of *E. rockii* were obtained (**Figure 1**). Primer design and PCR validation were performed based on the junctions of the graphical assembly results (**Supplementary Figures S1A–D**), which showed that the junctions and product lengths were as expected, as evidenced by the variable structure of the assembly results and other conformations of the mitochondria. Subsequently, by examining the connections between contigs and mapping withthird-generation long sequences, a relatively simplified primary conformation can be obtained (**Figure 2**; **Table 2**), with a total length of 549,593 bp, 514,248 bp, 481,576 bp, and 513,095 bp in *T. arundinaceum*, *M. sinensis*, *E. rockii*, and *N. porphyrocoma*, respectively.

**Table 2:** Mitogenome assembly of *Tripidium arundinaceum*, *Miscanthus sinensis*, *Erianthus rockii*, and *Narenga porphyrocoma*.

	*T. arundinaceum*	*M. sinensis*	*E. rockii*	*N. porphyrocoma*
Total length (bp)	549,593	514,248	481,576	513,095
Chromosome number	2	2	2	1
Chromosome 1 length (bp)	425,504	309,427	339,336	513,095
Chromosome 2 length (bp)	124,089	204,821	142,240	–
GC content	43.45%	43.28%	43.42%	43.64%
Gene number	62	60	64	71
Protein genes	34	32	36	36
rRNA genes	4	3	3	6
tRNA genes	24	25	25	29
Genes with introns	8	8	8	8

rRNA, ribosomal RNA.

A total of 51 unique genes were identified from the assembled mitogenome, comprising 32 PCGs, 16 transfer RNA (tRNA) genes, and three ribosomal RNA (rRNA) genes (**Table 3**). Notably, the gene copy numbers varied among four species: the *atp1* gene was duplicated in *T. arundinaceum*, *E. rockii*, and *N. porphyrocoma*, while the *atp8* gene exhibited duplication in *T. arundinaceum* and *N. porphyrocoma*. Additionally, *cox1* was found to be duplicated in *T. arundinaceum*, and *nad3* showed duplication in *E. rockii*. Eight genes were identified to contain one to four introns, including *ccmFc* (1), *cox2* (1), *nad1* (4), *nad2* (4), *nad4* (3), *nad5* (4), *nad7* (4), and *rps3* (1). It was noteworthy that in all four species, the exons of *nad1* and *nad5* were identified to be located on different chromosomes, requiring *trans*-splicing to generate complete transcripts.

**Table 3:** Gene content of mitogenome in *Tripidium arundinaceum*, *Miscanthus sinensis*, *Erianthus rockii*, and *Narenga porphyrocoma*.

Function of genes	Name of genes	*T. arundinaceum*	*M. sinensis*	*E. rockii*	*N. porphyrocoma*
ATP synthase	*atp1*	2	1	2	2
	*atp4*	1	1	1	2
	*atp6*	1	1	1	1
	*atp8*	2	1	1	2
	*atp9*	1	1	1	1
Cytochrome *c* biogenesis	*ccmB*	1	1	1	1
	*ccmC*	1	1	1	1
	*ccmFn*	1	1	1	1
	*ccmFc*	1	1	1	1
Cytochrome *c* oxidase	*cox1*	1	1	1	2
	*cox2*	1	1	1	1
	*cox3*	1	1	1	1
NADH dehydrogenase	*nad1*	1	1	1	1
	*nad2*	1	1	1	1
	*nad3*	1	1	2	1
	*nad4*	1	1	1	1
	*nad4L*	1	1	1	1
	*nad5*	1	1	1	1
	*nad6*	1	1	1	1
	*nad7*	1	1	1	1
	*nad9*	1	1	1	1
Maturases	*matR*	1	1	1	1
Transport membrane protein	*mttB*	1	1	1	1
Ubiquinol cytochrome *c* reductase	*cob-1(ctyb)*	1	1	1	1
	*rps1*	1	1	1	1
	*rps2*	1	1	1	1
	*rps3*	1	1	1	1
	*rps4*	1	1	1	1
	*rps7*	1	1	1	1
	rps12	1	1	2	1
	*rps13*	1	1	1	1
Large subunit of ribosome	*rpl16*	1	1	1	1
Ribosomal RNAs	*rrn5*	1	1	1	2
	*rrn18*	1	1	1	2
	*rrn26*	2	1	1	2
	*trnC*	2	1	1	1
	*trnD*	1	1	1	2
	*trnE*	1	1	1	2
	*trnF*	3	3	4	3
	*trnH*	1	1	2	1
	*trnK*	1	1	1	1
	*trnM*	4	4	4	6
	*trnN*	2	1	1	2
	*trnP*	2	2	2	2
	*trnQ*	1	2	1	1
	*trnS*	1	1	1	2
	*trnS1*	1	1	2	1
	*trnS2*	1	1	1	1
	*trnV*	1	2	1	1
	*trnW*	1	1	1	1
	*trnY*	1	2	1	2

### 3.2 Chloroplast-derived sequence analysis

During the evolution of mitochondria, chloroplast fragments were transferred to the mitogenome (Wang et al., 2007). Approximately 5%–10% of the sequences in mitogenome that can be identified as homologs are derived from the chloroplast genome (Rodríguez-Moreno et al., 2011). In this study, the chloroplast genomes of four species were reassembled and annotated based on PacBio HiFi reads, and then the transfer sequences between mitochondrial and chloroplast genomes were analyzed.

**Figure 3:**
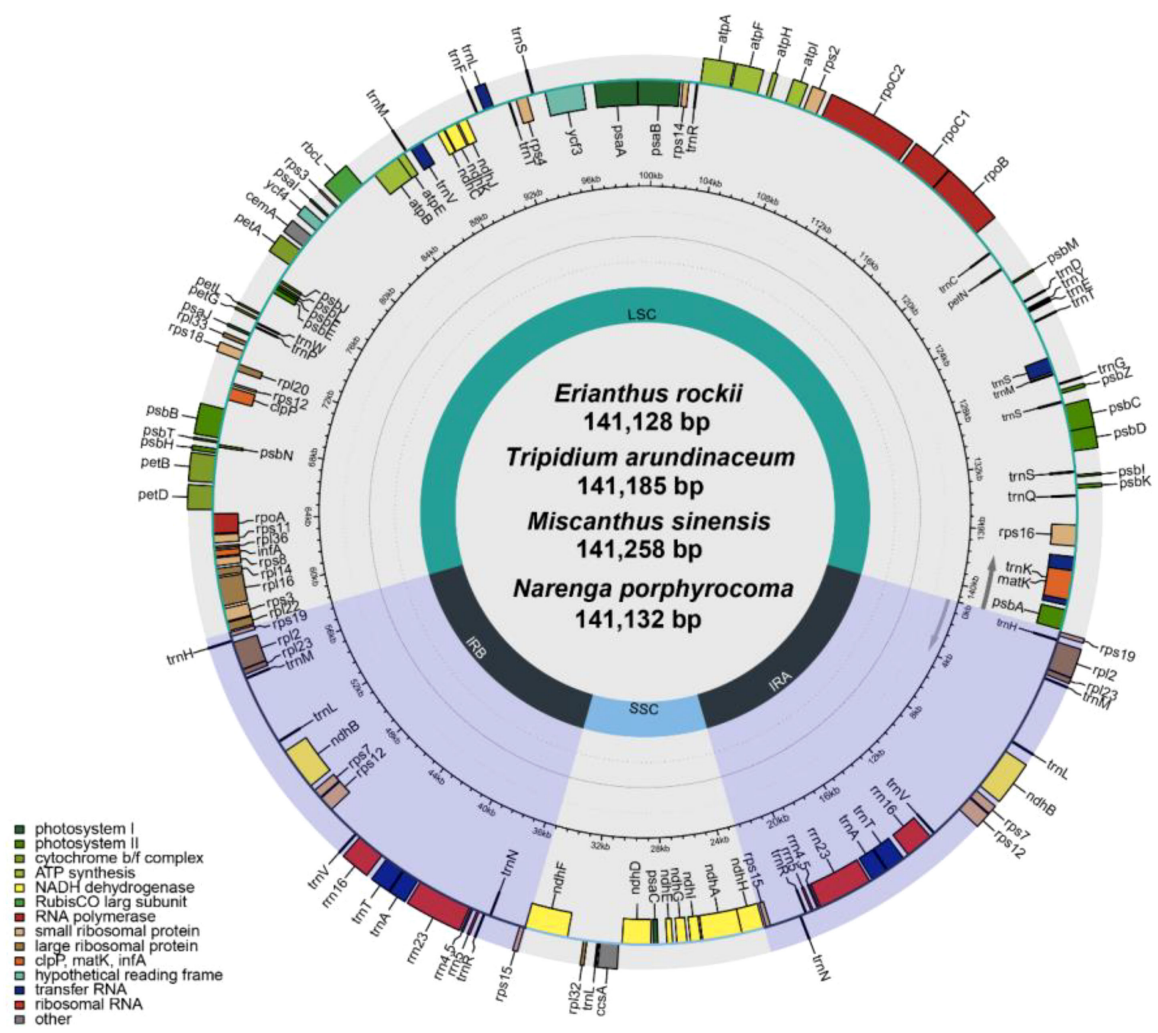
Gene map of the chloroplast genome of four Saccharinae species.

The four completed chloroplast genomes ranged in length from 141,128 bp to 141,258 bp with two inverted repeat regions (IRA and IRB), separating the large single-copy (LSC) and small single-copy (SSC) regions (**Figure 3**). Gene annotation showed that there were 130 genes in all assembled chloroplast (cp) genomes, including 83 protein-coding genes (74 single-copy PCGs; two copies of *ndhB*, *rps12*, *rps19*, *rpl2*, *rpl23*, *rps7*, and *rps15* in inverted repeat regions), 38 tRNA genes (two copies of *trnA-UGC*, *trnH-GUG*, *trnI-CAU*, *trnV-GAC*, *trnI-GAU*, *trnA-UGC*, *trnR-ACG*, *trnN-GUU*, and *trnL-CAA* in inverted repeat regions), and eight rRNA genes (two copies of *rrn23*, *rrn4.5*, *rrn5*, and *rrn16* in inverted repeat regions) (**Supplementary Table S5**). The results of reassembly by long read and annotation were similar to previously published second-generation sequencing assembled chloroplast genomes of the Saccharinae (Li et al., 2022).

The total fragment lengths of the MTPTs of *T. arundinaceum*, *M. sinensis*, *N. porphyrocoma*, and *E. rockii* were 37,559 bp, 55,349 bp, 40,117 bp, and 37,541 bp, respectively (**Supplementary Tables S6–9**). There were 43, 52, 44, and 43 plastid-derived sequences identified, accounting for 6.83%, 10.07%, 7.30%, and 6.83% of the respective mitochondrial DNA (mtDNA), and 26.60%, 39.18%, 7.30%, and 6.83% of the respective chloroplast DNA (cpDNA), respectively (**Table 4**). In *M. sinensis*, the transfer length, quantity, and proportion of MTPT fragments were the highest among all organelle genomes. The total length of MTPT fragments in *Saccharum* was approximately 3 kb, which was approximately 8% and 25% of the length of the mitochondrial and chloroplast genomes, respectively (Li et al., 2024).

**Table 4:** Chloroplast genome and MTPT information of *Tripidium arundinaceum*, *Miscanthus sinensis*, *Erianthus rockii*, and *Narenga porphyrocoma*.

Species	cp genome length (bp)	MTPT length (bp)	MTPT number	MTPT percent (in mtDNA)	MTPT percent (in cpDNA)
*T. arundinaceum*	141,185	37,559	43	6.83%	26.60%
*M. sinensis*	141,258	55,349	52	10.07%	39.18%
*E. rockii*	141,128	40,117	44	7.30%	28.43%
*N. porphyrocoma*	141,132	37,541	43	6.83%	26.60%

### 3.3 Phylogenetic analysis

**Figure 4:**
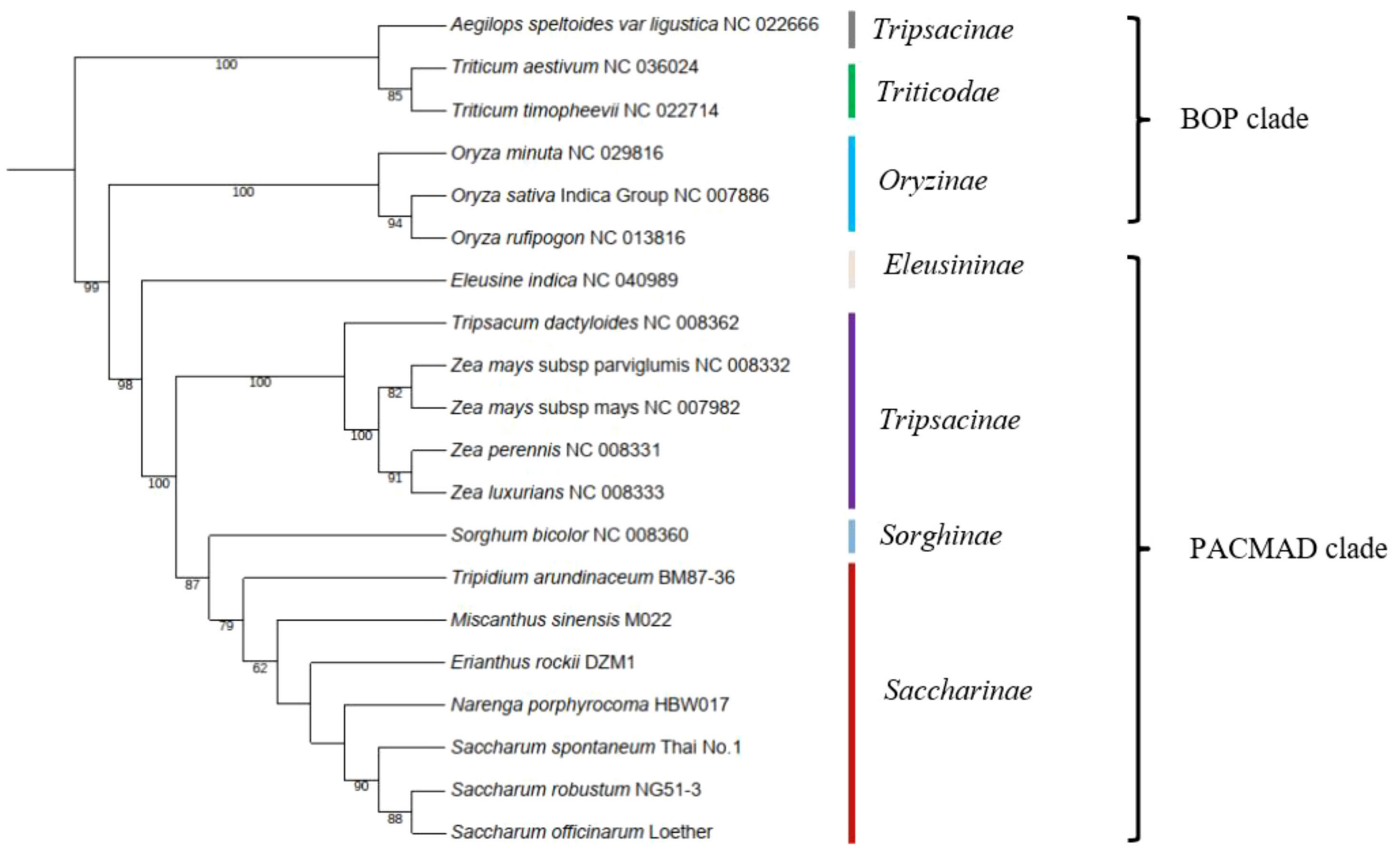
Phylogenetic tree of 20 species in BOP clade and PACMAD clade. The number at each node was the bootstrap probability.

Mitochondrial genes are a valuable source of information for phylogenetic analyses at large-scale taxonomic levels due to their low substitution rates. To determine the phylogenetic position of Saccharinae, we used 13 conserved mitochondrial PCGs (*ccmC*, *cox1*, *cox2*, *cox3*, *cob*, *matR*, *nad3*, *nad4L*, *nad9*, *rp3*, *rps4*, *rps7*, and *rps12*) shared in 20 Poaceae mitogenomes (**Figure 4**). Based on a relatively high support rate and the classification outlined by the Angiosperm Phylogeny Group (APG IV), Saccharinae belonged to the PACMAD clade in Poaceae. The four genera, *Tripidium*, *Miscanthus*, *Erianthus*, and *Narenga* from the core “*Saccharum* complex”, was monophyletic as sister to *Saccharum*. *Saccharum* spp. were clustered into the same clades, and *N. porphyrocoma* was the most closely related to the *Saccharum* genus, followed by *E. rockii*. *T. arundinaceum* and *M. sinensis* were not closely related to *Saccharum*. The phylogenetic relationships of species in the core “*Saccharum* complex” were consistent with previous chloroplast-based phylogenies (Li et al., 2022).

### 3.4 Collinearity analysis

**Figure 5:**
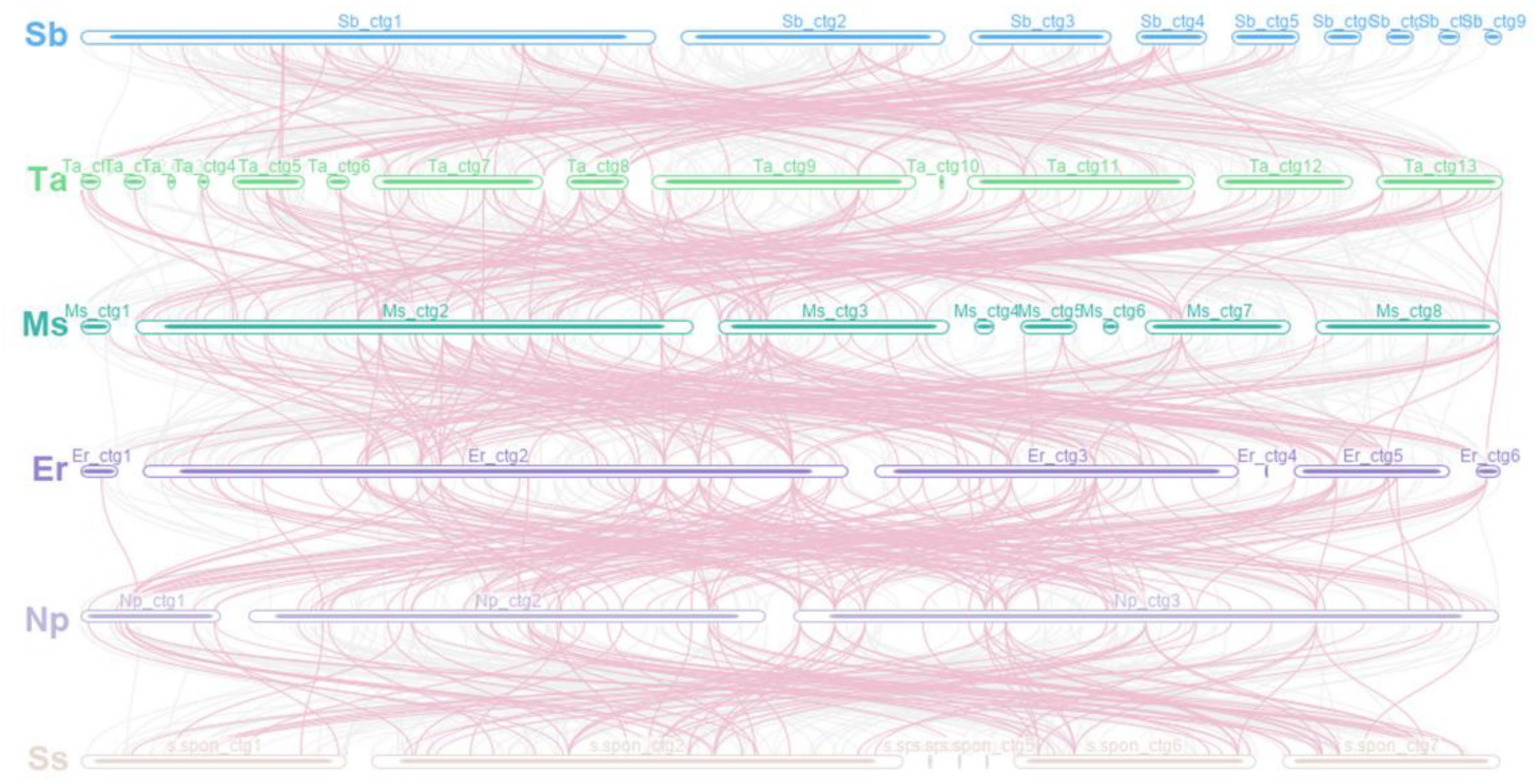
Mitogenome synteny. Bars indicate the mitogenomes, and the lines show the homologous sequences between the adjacent species. The pink line represents the sequence of inheritance between the six species. Sb, *Sorghum bicolor*; Ta, *Tripidium arundinaceum*; Ms, *Miscanthus sinensis*; Er, *Erianthus rockii*; Np, *Narenga porphyrocoma*; Ss, *Saccharum spontaneum*.

Sequence transfer between species was also explored to understand which sequences were retained in the mitochondrial genome during evolution and how these sequences were recombined between species. There were collinear segments with a total length of 335,303 bp, 392,247 bp, 360,963 bp, 420,218 bp, and 433,309 bp for *S. bicolor*–*T. arundinaceum* (350 fragments), *T. arundinaceum*–*M. sinensis* (369 fragments), *M. sinensis*–*E. rockii* (334 fragments), *E. rockii*–*N. porphyrocoma* (260 fragments), and *N. porphyrocoma*–*S. spontaneum* (277 fragments), respectively (**Figure 5**). Collinear sequences over 10 kb in length were retained in each species, and short collinear sequences were often lost after species divergence. In the graph assembly results, short collinear sequences were often nodes connected between long and repetitive contigs, which may have played a role in the conformation of the species but were not retained during evolution.

**Figure 6:**
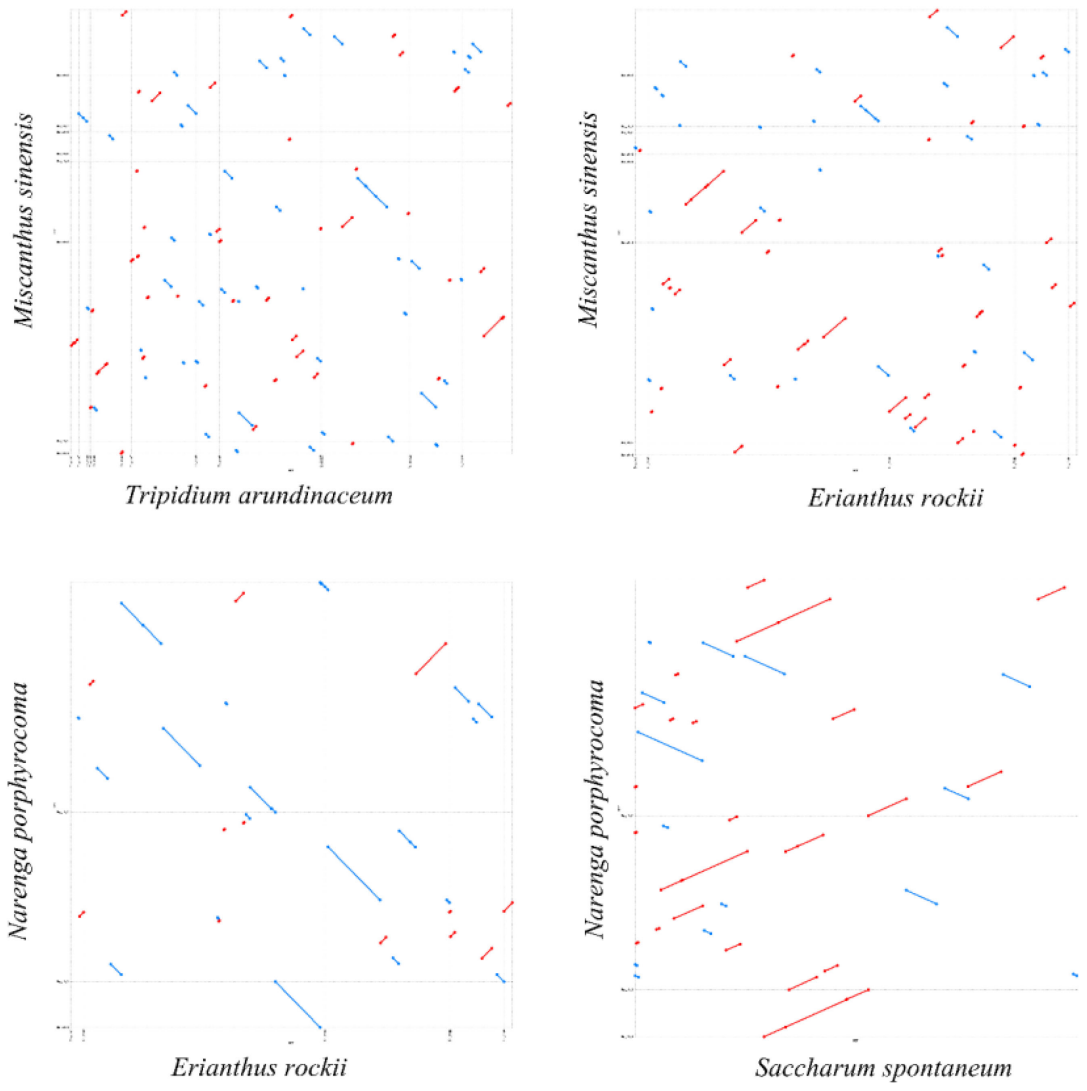
Dot-plot analysis. The blue and red lines represent reverse and forward sequences, respectively. Dot plot of *Tripidium arundinaceum* with *Miscanthus sinensis* is shown in the upper left corner, and the rest are dot plots of *M. sinensis* with *Erianthus rockii*, *E. rockii* with *Narenga porphyrocoma*, and *N. porphyrocoma* with *Saccharum spontaneum*.

Based on the phylogenetic relationships, we performed a dot-plot analysis of two species with close phylogenetic relationships. The results showed numerous collinear blocks in *T. arundinaceum*–*M. sinensis* and *M. sinensis*–*E. rockii*, but all were short and fragmented. However, long collinear blocks were found in both *E. rockii*–*N. porphyrocoma* and *N. porphyrocoma*–*S. spontaneum*. The relatively short divergence time between these two species may not have led to a large-scale reorganization of the mitogenome, and large collinear blocks are still retained between the two species (**Figure 6**). The mitogenomes in the subtribe taxonomic level had undergone extensive genomic rearrangements with closely related species, and the mitogenome was extremely not conserved in structure.

### 3.5 Gene duplication and loss, and characteristic difference of mitogenomes

**Figure 7:**
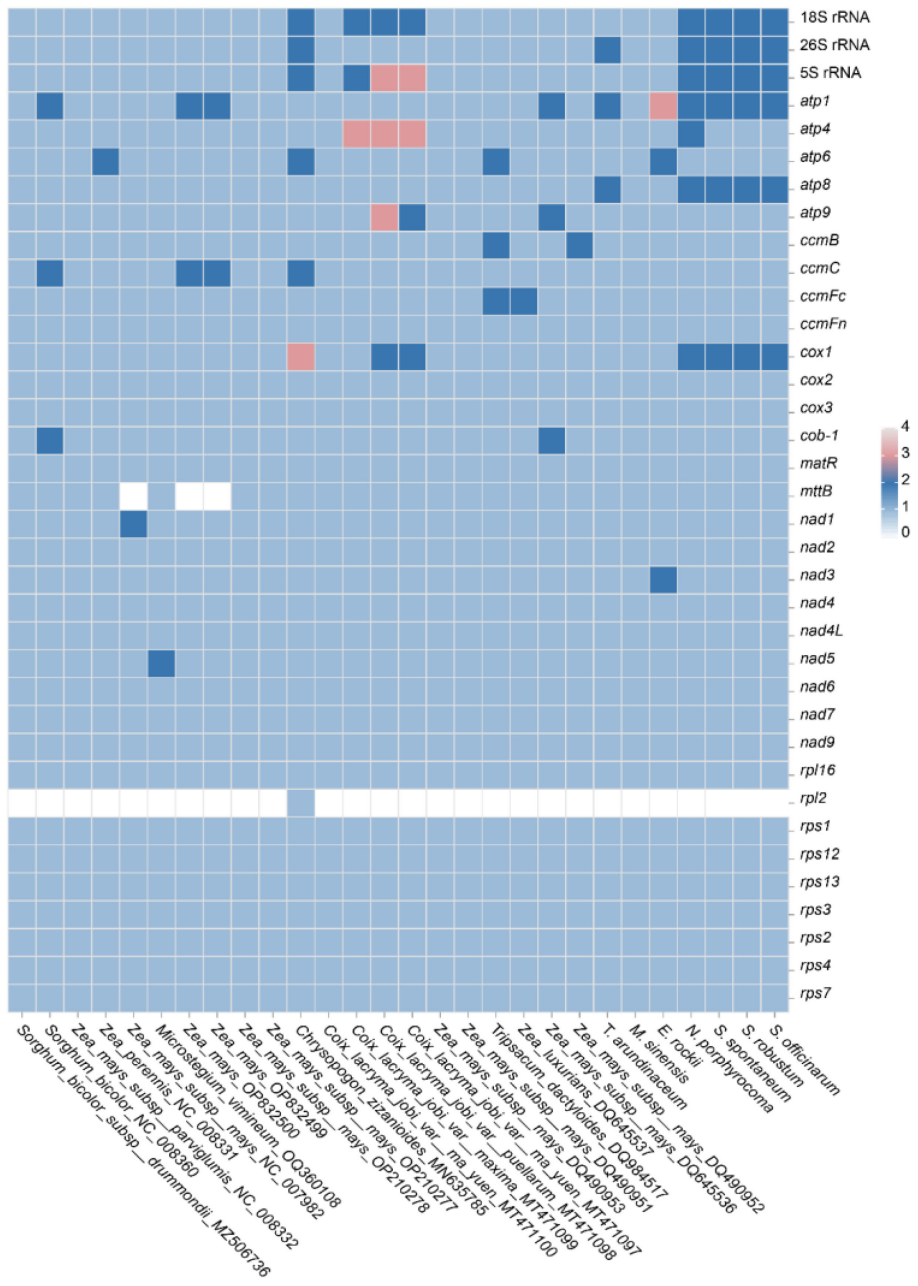
Gene duplication and loss in Andropogoneae species.

Gene duplication and loss often occur during the evolution of plants, and the genes that are retained by duplication are crucial for normal life activities. Therefore, gene duplication and loss in the Andropogoneae species were compared here (**Figure 7**). For the mitochondrial PCGs, it was found that the ATP synthase genes and the cytochrome *c* synthesis gene were not lost with strong conservation. However, the succinate dehydrogenase proteins (*sdh3* and *sdh4*) were found lost in Poaceae with high volatility and weak conservation during evolution. It was observed that genes within the ATP and COX family may exist in multiple copies in the Andropogoneae mitogenomes. For instance, *Z. mays* (OP832500) and *S. bicolor* (NC008360) had two copies of *atp1*; *Coix lacryma* (MT471100) harbored two copies of *atp4* and *atp9*; *C. zizanioides* (MN635785) exhibited a duplication of *atp6*. Furthermore, *cox1* was found in triplicate in *C. zizanioides* (MN635785) and duplicated in *C. lacryma* (MT471100). The *mttB* gene was not found in some accessions of *Zea*, which may be the impact of the incompetent annotation.

In Andropogoneae, most mitogenomes were published in the genus *Zea*, followed by genera *Coix*, *Saccharum*, and *Sorghum*. The length of mitogenomes ranged from 449,028 bp (*S. bicolor* subsp. *drummondii*, MZ506736.1) to 739,719 bp (*Z. mays* subsp. *mays* genotype CMS-C, DQ645536.1), with an average length of approximately 500 kb (**Table 5**). The total lengths in *T. arundinaceum*, *M. sinensis*, *E. rockii*, and *N. porphyrocoma* were 549,593 bp, 514,248 bp, 481,576 bp, and 513,095 bp, respectively, which were close to the published mitochondrial genome lengths of Andropogoneae. The mitochondrial genome lengths of species that have been domesticated in Andropogoneae, such as domesticated maize under the genus *Zea*, differ significantly from those of wild species (*Zea perennis* and *Zea luxurians*) of maize. In different accessions of *C. lacryma-jobi*, the length of the genome varied considerably, from 598,321 bp to 673,349 bp.

**Table 5:** Genome length and mitogenome transfer sequence of Andropogoneae species.

NCBI accession	Species	Genus	Genome length (bp)	MTMT number	MTMT length (bp)	MTMT percent
DQ645538.1	*Zea perennis*	*Zea*	570,354	298	129,437	22.69%
DQ645539.1	*Zea mays* subsp. *parviglumis*	*Zea*	680,603	582	329,614	48.43%
NC007982.1	*Z. mays* subsp. *mays*	*Zea*	569,630	404	127,771	22.43%
OP210278.1	*Z. mays* subsp. *mays*	*Zea*	553,762	388	110,681	19.99%
OP210277.1	*Z. mays* subsp. *mays*	*Zea*	557,050	386	117,279	21.05%
DQ490953.1	*Z. mays* subsp. *mays*	*Zea*	535,825	369	83,700	15.62%
DQ490951.2	*Z. mays* subsp. *mays*	*Zea*	557,162	388	117,343	21.06%
DQ645536.1	*Z. mays* subsp. *mays* genotype CMS-C	*Zea*	739,719	670	526,872	71.23%
DQ490952.1	*Z. mays* subsp. *mays*	*Zea*	701,046	592	379,011	54.06%
OP832500.1	*Z. mays*	*Zea*	565,153	376	122,311	21.64%
OP832499.1	*Z. mays*	*Zea*	567,959	354	123,723	21.78%
AY506529.1	*Z. mays*	*Zea*	569,630	404	127,771	22.43%
NC008333.1	*Zea luxurians*	*Zea*	539,368	333	62,398	11.57%
NC008362.1	*Tripsacum dactyloides*	*Tripsacum*	704,100	476	286,585	40.70%
MZ506736.1	*Sorghum bicolor* subsp. *drummondii*	*Sorghum*	449,028	276	78,815	17.55%
NC008360.1	*S. bicolor*	*Sorghum*	468,628	308	134,532	28.71%
MT411890.1	*Saccharum* hybrid cultivar FN15 chromosome 2	*Saccharum*	144,744	19	1,160	0.80%
MT411891.1	*Saccharum* hybrid cultivar FN15 chromosome 1	*Saccharum*	301,533	104	45,656	15.14%
MT821854.1	*Saccharum* hybrid × *Tripidium arundinaceum* chromosome 2	*Saccharum*	144,713	19	1,160	0.80%
MT821853.1	*Saccharum* hybrid cultivar × *T. arundinaceum* chromosome 1	*Saccharum*	300,848	124	45,656	15.18%
NC072666.1	*Microstegium vimineum*	*Microstegium*	478,010	226	90,643	18.96%
MT471098.1	*Coix lacryma-jobi* var. *puellarum*	*Coix*	673,349	378	158,499	23.54%
MT471100.1	*C. lacryma-jobi* var. *ma-yuen*	*Coix*	598,321	345	45,507	7.61%
MT471097.1	*C. lacryma-jobi* var. *ma-yuen*	*Coix*	660,150	370	134,084	20.31%
MT471099.1	*C. lacryma-jobi* var. *maxima*	*Coix*	639,298	354	148,704	23.26%
NC056367.1	*Chrysopogon zizanioides*	*Chrysopogon*	551,622	394	222,983	40.42%
In this study	*Erianthus rockii*	*Erianthus*	481,576	313	41,217	8.56%
In this study	*Tripidium arundinaceum*	*Tripidium*	549,593	318	48,805	8.88%
In this study	*Narenga porphyrocoma*	*Narenga*	513,095	322	159,697	31.12%
In this study	*Miscanthus sinensis*	*Miscanthus*	514,248	333	26,026	5.06%

Variations in mitochondrial genome sizes mainly result from the transfer of sequences from the nucleus and chloroplasts, as well as the expansion of repetitive sequences within the mitogenomes. We examined the Mitochondrion to Mitochondrion sequences (MTMTs) events and the proportion of the genome they occupied for species in Andropogoneae. *Z. mays* subsp. *mays* genotype CMS-C had not only the largest genome length but also the highest percentage of MTMTs (71.23%); meanwhile, the lowest percentage of MTMT sequences (0.80%) was found in chromosome 2 of *Saccharum* species.

## 4 Discussion

### 4.1 Mitogenome characterization in Saccharinae

Advances in sequencing and assembly strategies have made it possible for researchers to recover polymorphic conformations of plant mitochondria (Wang et al., 2024). In this study, we assembled the mitogenome of four species in Saccharinae first to provide a more comprehensive description of the mitogenome in Saccharinae, including basic mitochondrial features, structure, and homologous sequences.

In Saccharinae, the mitogenome length averaged approximately 500 kb. Our study revealed that *T. arundinaceum* had the longest mitogenome (549,593 bp). While the unique PCGs in Saccharinae mitogenomes were similar across the four species and *Saccharum*, variations in gene copy numbers were observed among them. Duplication of *cox1*, *atp1*, and *atp8* genes was found in *T. arundinaceum*; *atp1* and *nad3* genes in *E. rockii*; and *atp1* and *atp8* in *N. porphyrocoma*, but these genes were single-copy in *M. sinensis*. Within Andropogoneae, certain PCGs, such as those associated with ATP synthesis (*atp1*, *atp4*, and *atp8*) and the COX subunit complex (*cox1*), exhibited varying copy numbers from one to three. C4 plants in Andropogoneae, such as sorghum, maize, and sugarcane, are leading players in global agriculture and can survive in hot and dry conditions. Mitochondria play a crucial role in the internal regulation of organisms under harmful environmental conditions such as hypoxia and high temperature (Xiong et al., 2022). Through our analysis of gene duplications and losses in published Andropogoneae mitogenomes, we observed that most plants harbored multiple copies of ATP synthase and COX subunits, crucial for plant respiration. These findings offer insights into how C4 plants maintain cellular physiological functions in response to high temperatures.

The polymorphic structure of plant mitochondrial genomes often results in an uneven distribution of functional genes on each molecule, with many individual molecules lacking functional genes, and others having multiple copies of functional genes due to their presence in repetitive regions. For example, in the species within the *N. porphyrocoma* and *Saccharum* species, *cox1*, *atp8*, *rrn5*, *rrn18*, and *rrn26* all have two copies; *nad1* and *nad5* are usually present on different single molecules in the genomes of the Saccharinae, which are assembled into functional transcripts by *trans*-spicing. Single molecules lacking functional genes are often shown in graphical results as nodal molecules connecting long contigs, and they may have an important role in shaping the conformation of mitogenomes. We compared mitogenome synteny among six species, sorghum, *T. arundinaceum*, *M. sinensis*, *E. rockii*, *N. porphyrocoma*, and *S. spontaneum*, to identify transfer sequences among them. We found that most transfer sequences were long contigs and that short contigs (i.e., single molecules lacking functional genes) were rarely transferred between species, suggesting that they play a role in these species and that these sequences were not homologous by disrupted recombination after species formation.

### 4.2 Mitogenome evolution in Saccharinae

Most land plant mitogenomes analyzed to date can be represented as a single circular chromosome (called the major cyclic chromosome or master circle) and a set of secondary chromosomes (called the minor cyclic chromosome), which arise by active recombination of large direct repeats (Wang et al., 2024). Few comparative analyses of plant mitochondrial genes have so far revealed the relationship between species evolution and mitochondrial conformation. The simplest structures of the mitochondrial genomes of *Saccharum robustum* and *S. officinarum* in the genus *Saccharum* can be described as two-ring structures (300 kb and 144 kb), and the structure of the *S. spontaneum* as a 380-kb linear structure and a 100-kb-ring structure (Li et al., 2024). The diploid *N. porphyrocoma*, closely related to *Saccharum*, exhibited a single cyclic structure. Its secondary conformation can generate substructures of 225 kb and 288 kb, whose secondary conformation allowed the generation of 225 kb and 288 kb substructures, respectively. In *T. arundinaceum*, there was no connection between chromosome 1 (425 kb) and chromosome 2 (124 kb), similar to that in *S. robustum* and *S. officinarum*. However, the linear structures of *M. sinensis* and *E. rockii* can form a closed structure in the graph-based assembly. During speciation, the conformation of plant mitochondria may have undergone fusion and division to accommodate species formation.

Many studies have shown that mtDNA evolves faster in structure but slower in sequence change compared to cpDNA or nuclear DNA, making mtDNA a good tracer for genome evolution (Hu et al., 2023). Changes in gene copy number are common in plant mitochondria, and whole-genome duplication (WGD) events occurring in plant nuclear genomes can affect the relative copy numbers of nuclear, mitochondrial, and chloroplast genomes (Zwonitzer et al., 2024). Increases in organelle genome copy number represent a common response to polyploidization, suggesting that maintenance of nuclear stoichiometry is an important aspect of establishing polyploid lineages.

In the evolution of species, understanding how nucleoplasmic equilibrium evolves after polyploidization requires studying the exchange between mitochondrial and nuclear genomes of their diploid ancestors (Korpelainen, 2004). The ploidy levels of species within the Saccharinae vary significantly, with most of the close relatives of sugarcane being diploid (*N. porphyrocoma*, *M. sinensis*, and *E. rockii* in this study are diploid, while *T. arundinaceum* is tetraploid), some wild sugarcane species ranging from tetraploid to octoploid (Zhang et al., 2022), and cultivated sugarcane species being over octoploid (Zhang et al., 2019). Chromosome-level genome assemblies have been recently published for the genera *Erianthus* (*Erianthus rufipilus*) (Wang et al., 2023) and *Saccharum* (Healey et al., 2024). The structure and copy number of the mitogenome are closely associated with the balance of genetic material within the cell during polyploidization (Zwonitzer et al., 2024), but the evolutionary mechanisms of the mitochondrial genome following nuclear genome polyploidization remain to be studied. The mitogenomes of the four Saccharinae species assembled and analyzed in this study lay the groundwork for exploring nucleoplasmic interactions between the mitogenome and nuclear genome during WGD events in polyploidization within the Saccharinae.

## 5 Conclusions

In this study, we assembled graph-based mitochondrial genomes of four Saccharinae species (*T. arundinaceum*, *E. rockii*, *M. sinensis*, and *N. porphyrocoma*) closely related to sugarcane and provided a more complete characterization of the mitochondrial genomes instead of the “master circular” structure. Second, we performed a comparative mitogenome analysis of Saccharinae species, including characterization, transfer sequence (MTPTs and MTMTs), collinear sequence, phylogenetic analysis, and gene duplication/loss. Our results provided the mitochondrial genomes of four *Saccharum* complex species, enriching the mitochondrial genomic resources of the Saccharinae. Second, it provided new insights into the evolution of mitochondrial genomes at low taxonomic levels. Finally, it facilitated our understanding of organelle evolution in the highly polyploid *Saccharum* genus.

## Data availability statement

The datasets presented in this study can be found in online repositories. The names of the repository/repositories and accession number(s) can be found in the article/**Supplementary Material**. The data presented in the study are deposited in online repository, accession number *Narenga porphyrocoma* (PP551632.1), *Tripidium arundinaceum* (PP664562.1, PP664563.1), *Miscanthus sinensis* (PP665700.1, PP665701.1), and *Erianthus rockii* (PP622780.1, PP622781.1). The raw data used in this work were deposited at National Center for Biotechnology Information (NCBI), BioProject: PRJNA1097978.

## Author contributions

SL: Data curation, Investigation, Writing – original draft, Writing – review & editing. CY: Writing – review & editing, Conceptualization, Data curation. ZW: Data curation, Formal analysis, Writing – review & editing. CX: Writing – review & editing. GZ: Writing – review & editing. YH: Writing – review & editing. BZ: Conceptualization, Data curation, Formal analysis, Funding acquisition, Investigation, Methodology, Project administration, Resources, Software, Supervision, Validation, Visualization, Writing – review & editing. SZ: Writing – review & editing. YG: Writing – review & editing. WZ: Writing – review & editing. WD: Funding acquisition, Project administration, Resources, Writing – review & editing. XY: Conceptualization, Funding acquisition, Writing – original draft, Writing – review & editing.
