# Supplementary material for: Assembly and comparative genome analysis of four mitochondrial genomes from Saccharum complex species
Source: Front Plant Sci. 2024 Jul 19;15:1421170. doi: 10.3389/fpls.2024.1421170 (PMC11294102; doi:10.3389/fpls.2024.1421170)
Supplement: Supplementary file 1 [file Table_1.docx]

# **Supplementary Information**


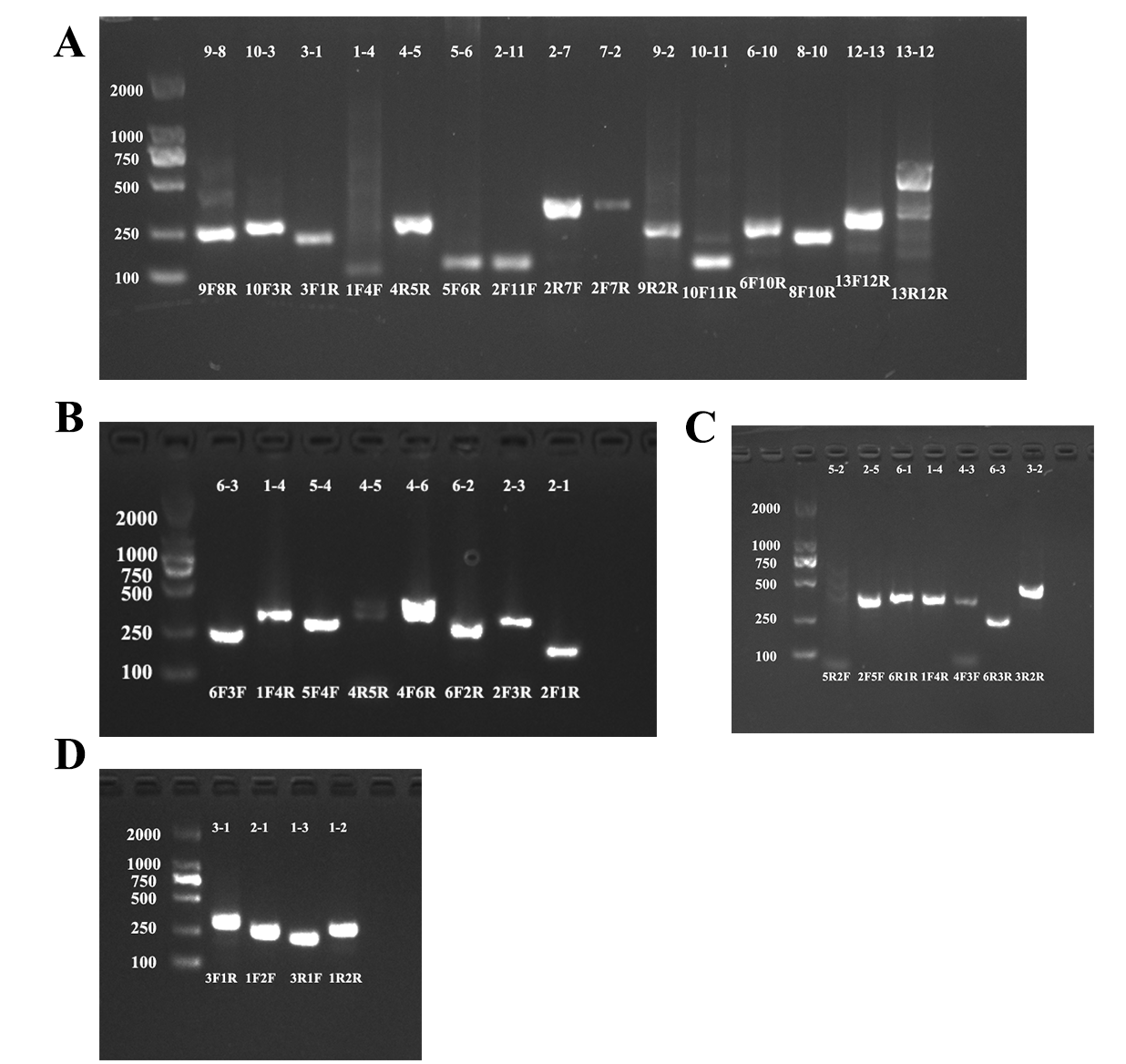


**Figure S1 PCR amplification to verify linkages junctions of four accessions. The numbers below each lane of the gel referred to linkages spanned by the primers in respect to the contigs in Figure 1. The numbers above each lane was the contigs connections in Figure 1. (a) PCR verification of possible connections in** ***T. arundinaceum* (accession: BM87-36); (b) PCR verification of possible connections in** ***E. rockii* (accession: DZM1) ;(c) PCR verification of possible connections in** ***M. sinensis*** **(accession: M022); (d) PCR verification of possible connections in** ***N. porphyrocoma* (accession: HBW017)*.***
